# The minimum required overlap length for tendon transfer A biomechanical study on human tendons

**DOI:** 10.1371/journal.pone.0289650

**Published:** 2023-08-04

**Authors:** Nikolaus Wachtel, Marc A. Englbrecht, Carina Micheler, Jan J. Lang, Rainer Burgkart, Riccardo E. Giunta, Christina J. Wilhelm

**Affiliations:** 1 Division of Hand, Plastic and Aesthetic Surgery, University Hospital, LMU Munich, Munich, Germany; 2 Women’s Clinic Dr. Geisenhofer, Munich, Germany; 3 Department of Orthopaedics and Sports Orthopaedics, Klinikum Rechts der Isar, TUM School of Medicine, Technical University of Munich, Munich, Germany; 4 Institute for Machine Tools and Industrial Management, TUM School of Engineering and Design, Technical University of Munich, Munich, Germany; 5 Chair of Non-Destructive Testing, TUM School of Engineering and Design, Technical University of Munich, Munich, Germany; 6 Maria-Theresia-Klinik, Academic Teaching Hospital of Ludwig-Maximilians University Munich, Munich, Germany; Universidade de Trás-os-Montes e Alto Douro: Universidade de Tras-os-Montes e Alto Douro, PORTUGAL

## Abstract

In tendon transfer surgeries sufficient stability of the tenorrhaphy is essential. In addition to the choice of a suitable technique, adequate overlap of donor and recipient tendons must be ensured. The aim of this study was to investigate the tensile strength with regard to tendon overlap of a recently published tenorrhaphy, termed Woven-Fridén (WF) tenorrhaphy, which displayed higher tensile strength and lower bulk when compared to the established Pulvertaft technique. For this purpose, WF tenorrhaphies with 1.5 cm, 2 cm, and 3 cm tendon overlap were performed and subsequently tested for different biomechanical properties by tensile testing. Among others, the parameters of ultimate load and stiffness were collected. Native tendons served as controls. A formula was derived to quantify the relation between tendon overlap and ultimate load. We observed that sufficient tensile strength (mean ultimate load of 217 N) is already given with a 2 cm tendon overlap. In addition, with more than 3 cm overlap length only little additional tensile strength is to be expected as the calculated ultimate load of 4 cm overlap (397 N) is approaching the plateau of the maximal ultimate load of 435 N (native tendons).

## Introduction

Tendon transfer surgeries with subsequent side-to-side tenorrhaphies are typically used to restore limb function after muscle trauma or lesions of the central or peripheral nervous system [1–4]. Common examples include the treatment of patients with tetraplegia, with the goal to restore grasping movements of the hands as well as Achilles tendon reconstruction after repeated rupture [5–8]. Here, optimal stability of the tenorrhaphy is essential, allowing for successful immediate active mobilization after surgery. The established standard technique for a tendon transfer was first described by Pulvertaft (PT) et al [9]. We recently proposed a new technique, termed the Woven-Fridén suture (WF), which displayed significantly greater tensile strength, more effective force transmission and lower bulk than the PT tenorrhaphy as well as the side-to-side technique initially described by Fridén and colleagues [8, 10–12]. Concerning side-to-side tenorrhaphies, sufficient overlap of the donor and recipient tendon is essential for tensile strength [8]. Thus, a tendon overlap of at least 5 cm is recommended [8, 11]. However, this would require a total additional tendon length of 10 cm (i.e. 5 cm per tendon), which is rarely possible in clinical practice [13–15]. Accordingly, if the overlap length is too short, only limited stability is to be expected [8]. However, sufficient stability is a necessary precondition for early active mobilization of the limb [16–18]. If this cannot be ensured, a longer overall rehabilitation and a worse outcome of the intervention must be expected [8, 16, 19].

These guidelines predominantly rely on empirical clinical observations [8]. Although the above-mentioned studies made recommendations on overlap length in tenorrhaphy, no comparative studies of tenorrhaphy with different overlap were performed [8, 9, 11]. With respect to tendon transfer techniques, studies that further elucidate the relation between tendon overlap length and tensile strength are also lacking. Moreover, the high tensile strength of new tenorrhaphy techniques, in particular the WF technique, are likely to provide sufficient stability even for shorter overlaps [16]. Considering the effects of tendon overlap on the rehabilitation regime and the overall outcome, this study was designed to objectively quantify the relation between tendon overlap and tensile strength. By using a human tendon model, we set out to assess (1) the relation between tensile strength and tendon-tendon overlap in the WF tenorrhaphy as well as (2) determine the minimum overlap length for the WF technique at which sufficient tensile strength is provided to allow an early active mobilization protocol.

## Materials and methods

### Ethics statement

Ethical approval for this study was obtained and written from the Ethics Committee of the Medical Faculty of LMU Munich, Germany (approval number: 19–142). The tendons were harvested from body donors at the Anatomical Institute of the LMU Munich. These donors had stipulated during their lifetime that their bodies should be used postmortem for research and teaching purposes.

### Inclusion, exclusion and randomization

Superficial and deep flexor tendons of the upper extremity (FDS and FDP) from human body donors in Thiel fixation were harvested [11, 20]. Thiel fixation is used for lifelike preservation of whole bodies for anatomical preparation [20–22]. For this purpose, it is injected into the blood vessels. After the anatomical preparation, storage took place in a diluted version of the Thiel solution, Formalin was used for this purpose [20, 21, 23]. It is a partial component of Thiel solution [24, 25]. After harvesting, tendons were stored in formalin for two weeks. Block randomization was then used to determine which pairs of tendons were used in the experimental groups. The tendons were distributed in a balanced way among the groups of the experimental series [10].

### Tenorrhaphies

**Fig 1:**
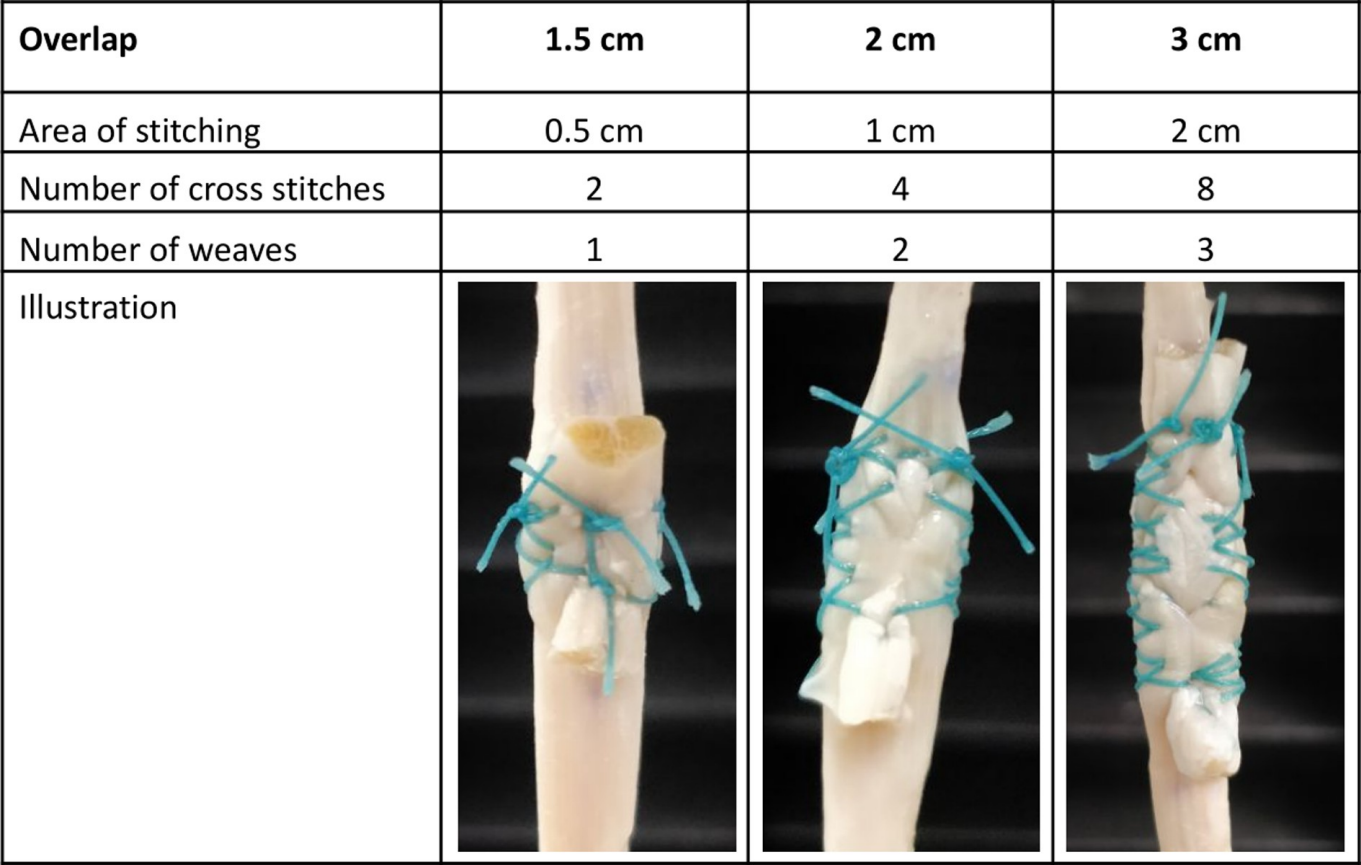
Tenorrhaphies using the Woven-Fridén technique on human tendons. Representation of specimens with variable overlap of the donor (top) and the recipient tendon (bottom) at 1.5 cm (left), 2 cm (middle) and 3 cm overlap (right) respectively.

WF tenorrhaphies were performed as previously described by Wilhelm et al. (**Fig 1**) [10]. Three experimental groups with different overlap lengths of 1.5 cm, 2 cm and 3 cm were compared. For the test groups with 2 cm and with 3 cm overlap 10 samples were tested, for the group with 1.5 cm overlap 9 tenorrhaphies were tested. Accordingly, a total of 29 samples were loaded until tensile failure. In addition, 10 native tendons served as control group and were tested by tensile testing.

The number of interlacing and the number of stitches were chosen according to the overlap length, proportional to the area of stitching (**Fig 1**). EthibondTM 3–0 (Ethicon, Inc. Somerville, NJ, USA) was used as suture material.

### Suture characteristics and tensile tests

The length of the overlap was measured with a digital caliper after completion of the tendon suture. During tensile testing, stiffness (resistance of the sutures to deformation), first failure load (first local maximum in the load-deformation curve) and ultimate load (force (N) reached at ultimate failure) were measured as previously described [10, 11, 26, 27].

All experiments were conducted according to a standardized protocol, similar to the protocols of previous studies [10–12, 26, 27]. All mechanical tests were performed with a calibrated tensile testing machine (Zwicki 1120, ZwickRoell GmbH & Co. KG, Ulm, Baden-Württemberg, Germany). Here, a preload of 2 N was applied and 5 preconditioning cycles were performed with a deformation of 5% of the inter-clamp distance [10, 11, 28]. Preconditioning was performed at a rate of 10 mm/min and then the tenorrhaphies were stretched to failure at a rate of 100 mm/min [10, 11, 27]. The results of the tensile tests were plotted on a force-deformation curve (TestXpert V12.0 ZwickRoell GmbH & Co. KG, Ulm, Baden-Württemberg, Germany). Using a custom developed MATLAB code (MATLAB R2017b, The MathWorks, Inc. Natick, MA, USA), load at first failure, ultimate load and stiffness were determined. The mean values and standard deviations were calculated for the different groups. The tensile tests were filmed with a Legria HF M31 video camera (Canon Co. Ltd., Ohta-ku, Tokyo, Japan) to document the nature of the failure [29, 30].

### Statistical analysis

Data are presented as mean values and standard deviation (SD). One-way analysis of variance (ANOVA), and then Tukey-Kramer multiple comparisons test were performed to evaluate the effects of different overlap lengths on repair stiffness, first failure load and ultimate load. A p-value of < 0.05 was considered statistically significant. The software used for the statistical analysis was GraphPad Prism 6 (GraphPad Software, Inc., San Diego, CA, USA).

### Formula derivation and extrapolation of further data sets

A formula was derived to describe the relation between overlap length and ultimate load: In a coordinate system where the x-axis represents the overlap length of tenorrhaphies and the y-axis represents the corresponding ultimate load, the resulting graph was plotted, where:

- ***f(x)*** corresponds to the ultimate load as a function of the overlap length.
- ***D***_***f***_ ***= [x***_***0***_***; ∞ [***is the definition range of the graph.
  - ***x***_***0***_ represents the beginning of the definition range, as no stitches can be applied below this threshold (in case of too little tendon overlap). The graph intersects the x-axis at *x*_*0*._
  - Theoretically, an infinite tendon overlap is conceivable. (Practically it is reduced as human tendons are limited in length).
- ***f(x) = -e***^***-x***^ represents the standard basic function for curve approximation. When *x* approaches infinity, *y* approaches an asymptote (initially *y = 0*).
- ***f***_***max***_ is the ultimate load of native tendons. At maximum overlap, the tenorrhaphies can withstand the same force as the native tendons (i.e. *f*_*max*_*(native)*) and therefore the graph approaches *y = f*_*max*_*(native)* as its asymptote.
- ***b*** is a factor describing the curvature behavior of the graph. *b* represents the overlap length at which the value of the ultimate load decreases by a factor of *1/e* compared to *f*_*max*_*(native)*.

Based on the above considerations, the graph is composed as follows (**formula 1**):

$$f(x)=\text{fmax}(\text{native})*\left(1-e^{-\frac{1}{b}*\left(x-x_{0}\right)}\right)$$

After determining the values of ultimate load for samples with different overlap lengths, the Curve Fitting Tool of the Matlab program was used to determine the shape of the curve (*b*) and x_0_.

## Results

### Ultimate load and stiffness for different tenorrhaphy-overlaps

**Fig 2:**
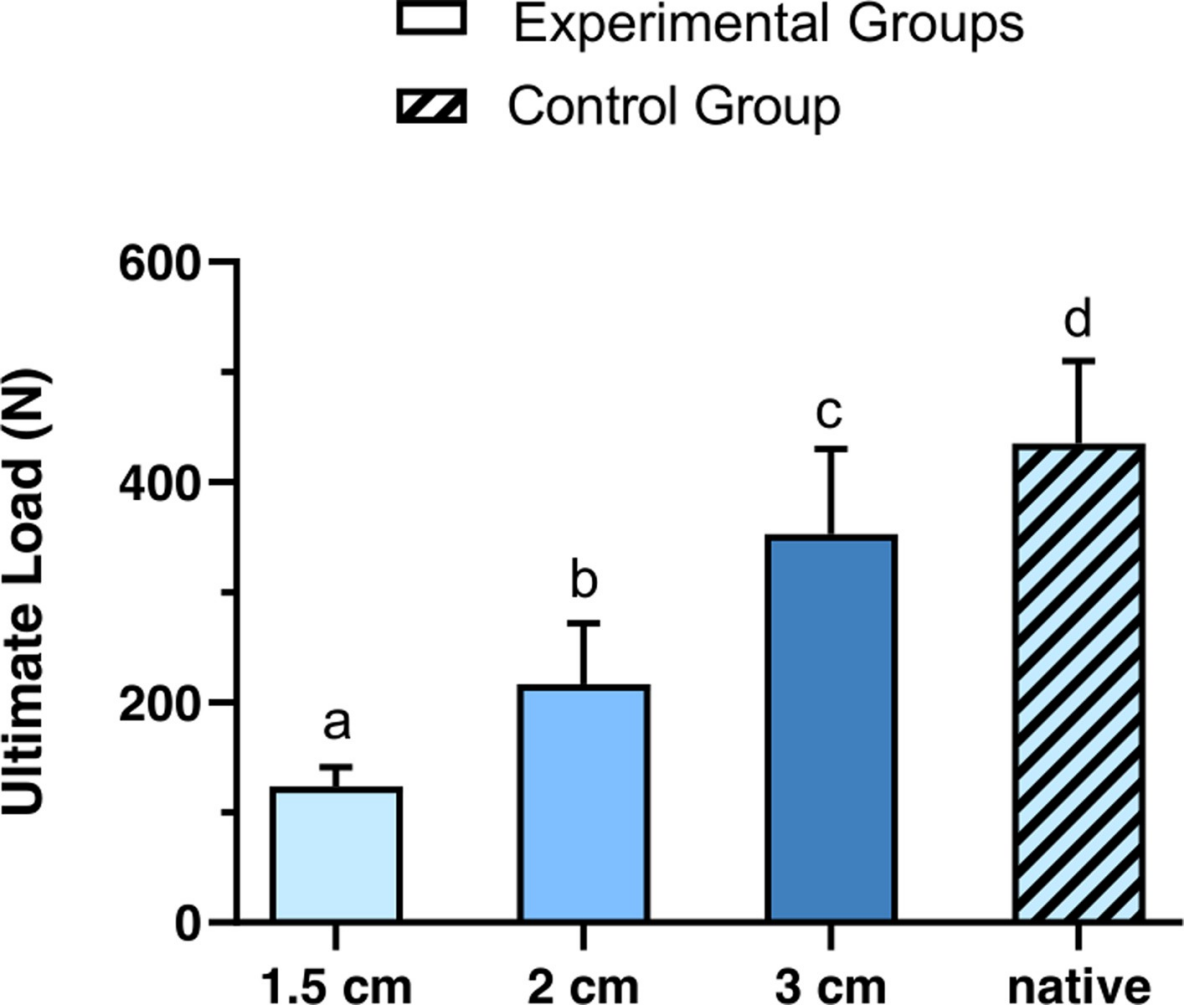
Comparison of the ultimate load of tenorrhaphies with 1.5 cm, 2 cm and 3 cm overlap (experimental groups, single-color bars) with native tendons (control group, crosshatched bar). Data are expressed as means, bars with standard deviations are indicated. Different superscripts indicate statistically significant differences among the groups (p < 0.05 for 1.5 cm vs. 2 cm and for 3 cm vs. native; p < 0.001 among the remaining groups). For the control group, 10 native tendons were tested. For the experimental groups with 2 cm and 3 cm overlap 10 samples were tested respectively, for 1.5 cm overlap 9 samples were tested.

All experimental groups showed statistically significant differences to each other in terms of ultimate load (all at least p < 0.05; **Fig 2**). The highest mean values in ultimate load were measured at 3 cm overlap (352.5 N (77.8 N)), followed by 2 cm (216.6 N (55.2 N)) and 1.5 cm overlap (118.5 N (14.9 N)). The ultimate load of native tendons (434.8 N (75.3 N)) showed statistically significant differences to all experimental groups (all at least p < 0.05).

**Fig 3:**
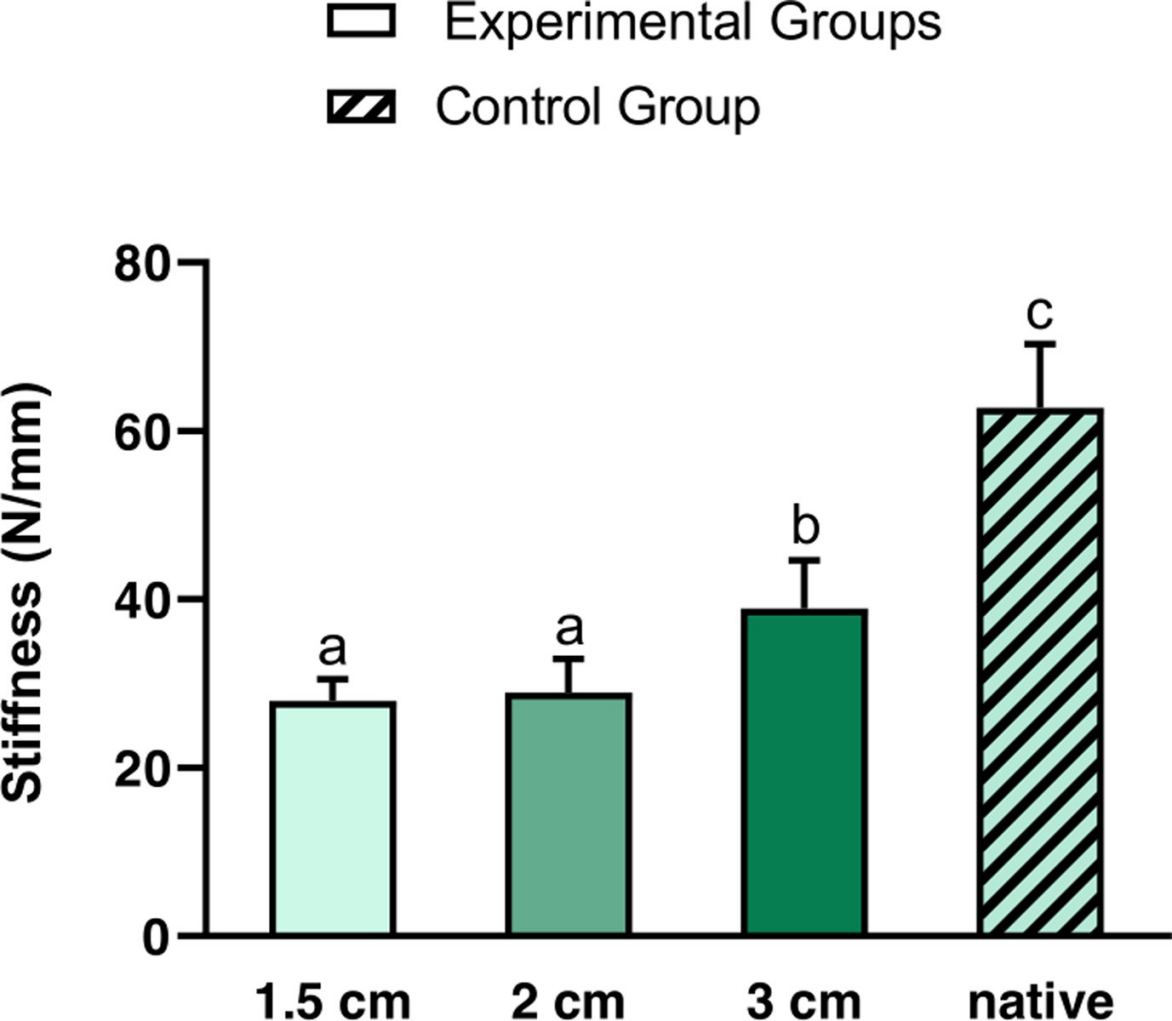
Comparison of the stiffness of tenorrhaphies with 1.5 cm, 2 cm and 3 cm overlap (experimental groups, single-color bars) with native tendons (control group, crosshatched bar). Data are expressed as means, bars with standard deviations are indicated. Different superscripts indicate statistically significant differences between groups at p < 0.001 and for 2 cm vs. 3 cm at p = 0.001. For the control group, 10 native tendons were tested. For the experimental groups with 2 cm and 3 cm overlap 10 samples were tested respectively, for 1.5 cm overlap 9 samples were tested.

Similarly, the stiffness (i.e. resistance to deformity) of the tenorrhaphies also increased with increasing overlap **(Fig 3)**. At 1.5 cm (27.9 N/mm (2.6 N/mm)) and at 2 cm (28.9 N/mm (4.0 N/mm)) the values were significantly lower than at 3 cm tendon overlap (38.9 N/mm (5.7 N/mm)) (p ≤ 0.001). Highest Stiffness was measured for native tendons (62.8 N/mm (7.6 N/mm)) with statistically significant differences to the three experimental groups at p < 0.001.

### Formula derivation to describe the relationship for tendon overlap and tensile strength

The measured overlap of the tenorrhaphies was lower after suturing was completed. In the experimental group with 1.5 cm overlap, the overlap decreased by approx. 1 mm, with 2 cm overlap by approx. 2 mm and with 3 cm overlap by approx. 3 mm. The range of overlap after suturing x_after_ was measured to be approx. 10% less than before x_before_ as described in **formula 2**:

$$x_{after\;}=0,9*x_{before\;}$$

In the following, x_after_ is equated with x. Based on the obtained results for ultimate load (**Fig 2**), the variables of formula 1 were determined using Matlab (b = 10.3 mm, x_0_ = 10.9 mm, Goodness of fit: R-square = 0.73). **This results in the following formula 3:**

$$f(x)=434.8N*\left(1-e^{-\frac{1}{10.3mm}*(x-10.9mm)}\right)$$

**Formula 3** can be used to extrapolate the expected average ultimate load values for untested overlap lengths (see **Table 1**). Subsequently, we added a corresponding safety factor for each group. The safety factor corresponds to the relative difference between the maximum force the FDP and FDS muscles can generate (mean of 69.3 N) and the tensile strength (ultimate load) of the tenorrhaphy [11, 31].

**Table 1:** Determined (bold) and extrapolated by formula 3 (italic) ultimate loads with corresponding safety factors for tenorrhaphies of variable overlap.

**Overlap (x**_**before**_ **)**	**1.5 cm**	**2.0 cm**	**3.0 cm**	*4*.*0 cm*	*5*.*0 cm*	*6*.*0 cm*
**Ultimate load (f**_**max**_ **)**	**123.3 N**	**216.6 N**	**352.4 N**	*396*.*8 N*	*418*.*9 N*	*428*.*2 N*
**Safety factor**	**1.8**	**3.1**	**5.1**	*5*.*8*	*6*.*1*	*6*.*2*

For the test groups with original tendon overlap x_before_ from 1.5 cm to 3.0 cm the experimentally determined mean ultimate loads were given. The ultimate load for 4.0 cm, 5.0 cm and 6.0 cm overlap was extrapolated using formula 3. The safety factor corresponds to the relative difference between the maximum force the FDP and FDS muscles can generate [11, 31].

## Discussion

**Fig 4:**
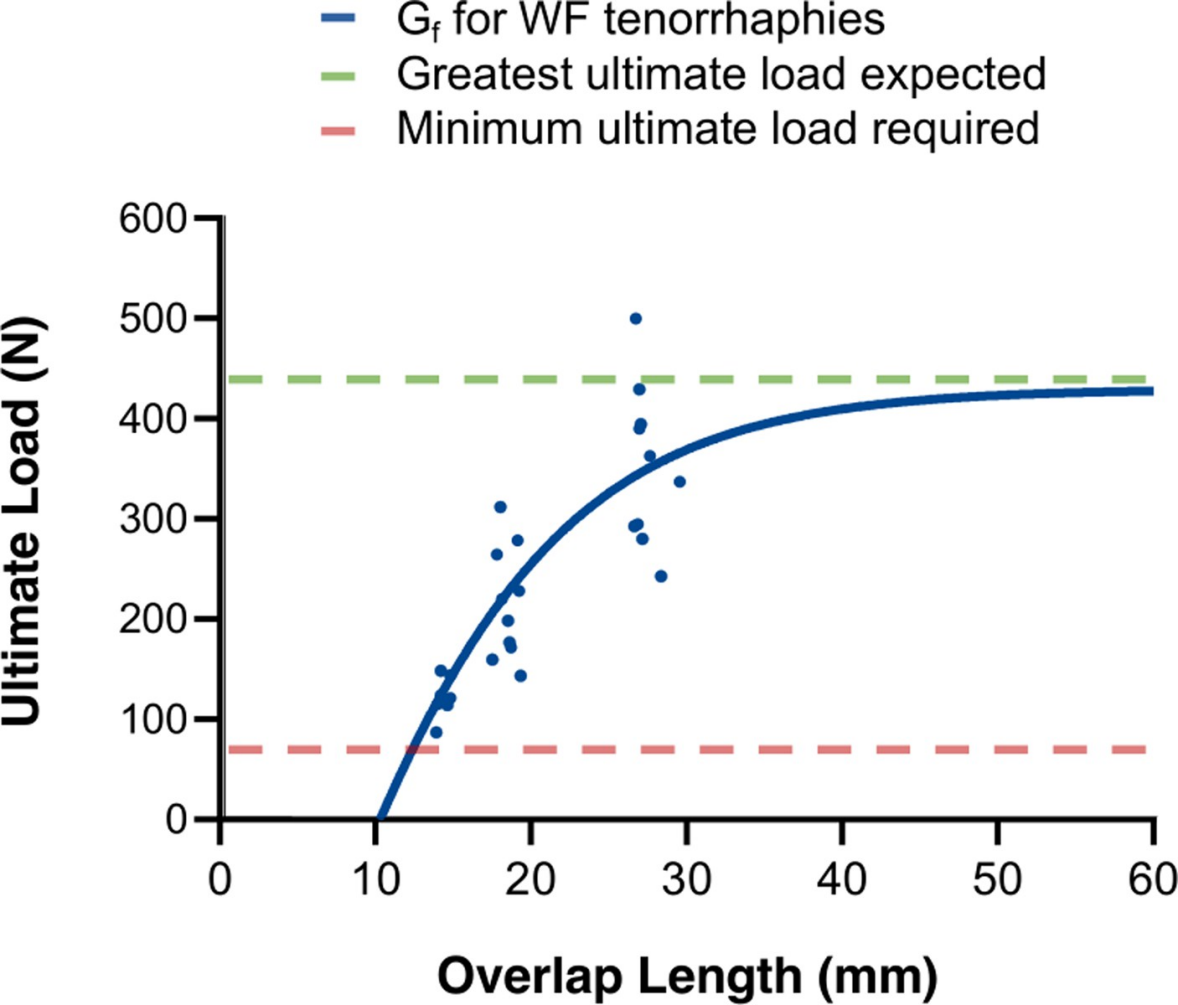
Load bearing capacity of tenorrhaphies in Woven-Fridén (WF) suture technique as a function of the overlap length of the tendons. The graph G_f_ (blue) illustrates the relation between overlap length in mm of WF tenorrhaphies (x-axis) and the corresponding ultimate load in N (y-axis). Each point in the coordinate system represents one sample (blue dots). The green line represents the greatest ultimate load expected (corresponding to the ultimate load of native tendons, i.e.control group). It can be approximated to 435 N. The red line represents the minimum required load capacity of the tenorrhaphies (corresponding to the maximum force expected by contraction of the flexor digitorum profundus (FDP) and flexor digitorum superficialis (FDS) muscles). It is given by Ward et al. as approximately 69 N [31]. The intersection of the graph at the x-axis (x0) represents the beginning of the definition range, as no WF tenorrhaphies can be performed below this threshold (in case of too little tendon overlap). Therefore, no load bearing capacity is to be expected at this point.

In this study we demonstrate that increasing the tendon overlap results in both higher ultimate load bearing capacity as well as stiffness (i.e. resistance to deformity) of WF tenorrhaphies (**Figs 2 and 3**). In order to comprehensively assess the correlation between ultimate load and tendon overlap, **formula 3** was derived, describing the relation between a targeted tendon overlap (independent variable) and a resulting ultimate load (dependent variable). To further illustrate this relation with regard to necessary load baring capacities seen in the clinical setting, the graph of the function is shown in **Fig 4**.

It is noticeable that with increasing overlap of the test groups, the differences in ultimate load decrease. When increasing the overlap from 2 cm to 3 cm, the ultimate load mounts by 136 N, resulting in an increase of the safety factor by 2 (**Fig 4 and Table 1**). When comparing 3 cm and 4 cm overlap, the gain in ultimate load is only 44.4 N, resulting in an increased safety factor by only 0.7.

With regard to the clinical setting, tendon length available for tenorrhaphy is limited. Increasing the overlap of the tenorrhaphy is associated with additional trauma [14]. A transfer that is too short may not ensure adequate stability. Decreased muscle excursion and function may occur [8, 13–15]. The results of this study demonstrate that only little additional stability is to be expected with an overlap greater than 3 cm. For example, at 4cm overlap, the expected ultimate load of 397 N is already approaching the plateau of the maximally expected ultimate load of 435 N that was observed in native tendons (**Fig 2**). More so, our results show that a WF tenorrhaphy with a 2 cm overlap may already provide sufficient stability **(Table 1).** Here, the safety factor is sufficiently large (on average 3.1), even when considering the standard deviation of samples measured (the weakest sample demonstrated an ultimate load of 145.4 N, resulting in a safety factor of 2.1). Accordingly, 2 cm can be recommended as the minimum required overlap.

When additionally regarding the stiffness of the WF sutures, the mean values increase with increasing overlap, as is to be expected. The comparison with the control group (native tendons) is particularly interesting: the stiffness of native tendons amounted to 62.8 N/mm and was thus about twice as high as the mean stiffness of all WF tenorrhaphies. The efficiency of force transmission is therefore significantly lower with tenorrhaphies than with native tendons **(Fig 3).** This may explain why tendon transfer regularly results in a reduction of muscle strength after surgery [4, 32].

This study was conducted *in vitro* and all experiments were performed on tendons collected postmortem. This is a limitation as our findings are therefore only an approximation to the situation *in vivo*. However, the proposed safety factor is likely to consider this limitation. Furthermore, it must be taken into account that fixation in Thiel solution/ formalin might alter the ultimate load or the stiffness of the tendons. Fewer sources of error would be expected in *in vivo* experiments. Based on our literature review, however, it can be assumed that the parameters of ultimate load as well as stiffness are not significantly changed by Thiel solution or formalin when compared to fresh tendons [22, 24, 25, 33, 34]. If anything, the fixation may cause a reduction in the ultimate load [22, 24]. Thus, the tenorrhaphies are likely to have even greater tensile strength *in vivo* than in our experiments.

In conclusion, this study demonstrates a clear relationship between tensile strength and tendon-tendon overlap in WF tenorrhaphies. Furthermore, we were able to define a minimum tendon-tendon overlap of 2.0 cm at which sufficient stability is to be expected. These findings should be verified in future studies *in vivo*.

## Supporting information

S1 Data Formula derivation. (DOCX)

S1 File Results. (DOCX)
