# Supplementary material for: The minimum required overlap length for tendon transfer A biomechanical study on human tendons
Source: PLoS One. 2023 Aug 4;18(8):e0289650. doi: 10.1371/journal.pone.0289650 (PMC10403071; doi:10.1371/journal.pone.0289650)
Supplement: S1 Data — (DOCX) [file pone.0289650.s001.docx]

**Supplementary Data – Formula Derivation**

First, the definition range *D_f_* of the graph *G_f_* is to be determined. Since there is a suture-free tendon end of 5 mm on both the donor and the recipient tendon, a first stitch would only be placed at an overlap of >10 mm. Thus, a minimum overlap of little more than 10 mm is necessary. The graph *G_f_* is therefore defined for the definition range *D_f_* (see below). It is assumed that the graph intersects the x-axis at the overlap of x ≈ 10.2 mm:

$$D_{f}= \left[ 10.2mm ; \infty\right[$$

Now the question arises how the graph *G_f_* behaves with an overlap of x → ∞. If the overlap of the tendons were infinitely long and thus the stability of the seam infinitely large, the sample would not tear in the area of the suture but in the area of the native tendon. The graph can therefore reach a maximum of the y-value, which represents the ultimate load of the native tendons. Accordingly, there is an asymptote *A_f_* that runs parallel to the x-axis, with *y = F_max_(native)*. The graph for *x → ∞* approaches this asymptote. For the ultimate load of the native tendons, 434.8 N was determined as the mean value. The following applies for the asymptote *A_f_*:

$$y=F_{max}(nativ)=434.8 N$$

Based on the described properties of the graph *G_f_*, it can most likely be described by an exponential function with a negative exponent:

$${f\left( x \right)= A*(1-e}^{-\frac{1}{b}*(x-x_{0)}})$$

Here, *A* corresponds to the y-value of the asymptote, hence the ultimate load of the native tendons *F_max_(native)* with approx. 434.8 N. *x_0_* is the intersection of the graph with the x-axis. The value *b* corresponds to the overlap length at which the value of the ultimate load has decreased by a factor of 1/e in relation to the maximum ultimate load. The Curve Fitting Tool of the Matlab program was used to determine the values *b* and *x_0_*. For this, all coordinates of the graph were entered (the x-values correspond to the measured overlaps of the tendons *x*, the y-values to the measured ultimate loads of the specimens *F_max_(x)*). For *b*, 10.3 mm is given, *x_0_* is approximated with 10.9 mm and thus corresponds approximately to the previously assumed beginning of the definition range.
