# Supplementary material for: The minimum required overlap length for tendon transfer A biomechanical study on human tendons
Source: PLoS One. 2023 Aug 4;18(8):e0289650. doi: 10.1371/journal.pone.0289650 (PMC10403071; doi:10.1371/journal.pone.0289650)
Supplement: S1 File — (DOCX) [file pone.0289650.s002.docx]

**Supplementary Data: Results**

| **Experimental Groups** | **1.5 cm** (n = 9) | **2 cm** (n = 10) | **3 cm** (n = 10) | **Native Tendons**  (n = 10) |
| --- | --- | --- | --- | --- |
| Overlapping length in mm | 14.3 (0.3)^a^ | 18.4 (0.6)^b^ | 27.2 (0.9)^c^ | - |
| Stiffness in N/mm | 27.9 (2.6)^a^ | 28.9 (4.0)^a^ | 38.9 (5.7)^b^ | 62.6 (7.6)^c^ |
| First Failure Load in N | 118.5 (14.9)^a^ | 214.3 (55.5)^b^ | 330.2 (73.0)^c^ | 433.7 (73.6)^d^ |
| Ultimate Load in N | 123.3 (17.7)^a^ | 216.6 (55.2)^b^ | 352.5 (77.8)^c^ | 434.8 (75.3)^d^ |

**Table 2. Comparison of the different characteristics of tenorrhaphies with three different overlap lengths: 1.5 cm (n = 9), 2 cm (n = 10), 3 cm (n = 10) and native tendons (n = 10).** The values are given as mean (SD). Different superscripts indicate statistically significant differences between groups at a p-value of at least < 0.05.
